# Supplementary material for: Association between oral microbiome and seven types of cancers in East Asian population: a two-sample Mendelian randomization analysis
Source: Front Mol Biosci. 2023 Nov 21;10:1327893. doi: 10.3389/fmolb.2023.1327893 (PMC10702768; doi:10.3389/fmolb.2023.1327893)
Supplement: Supplementary file 1 [file DataSheet1.ZIP › Supplementary Materials/MR plots for tongue/tongue═╝/Ovarian cancer/pheno.2184_to_ovarian cancer_leave_one_out.pdf]

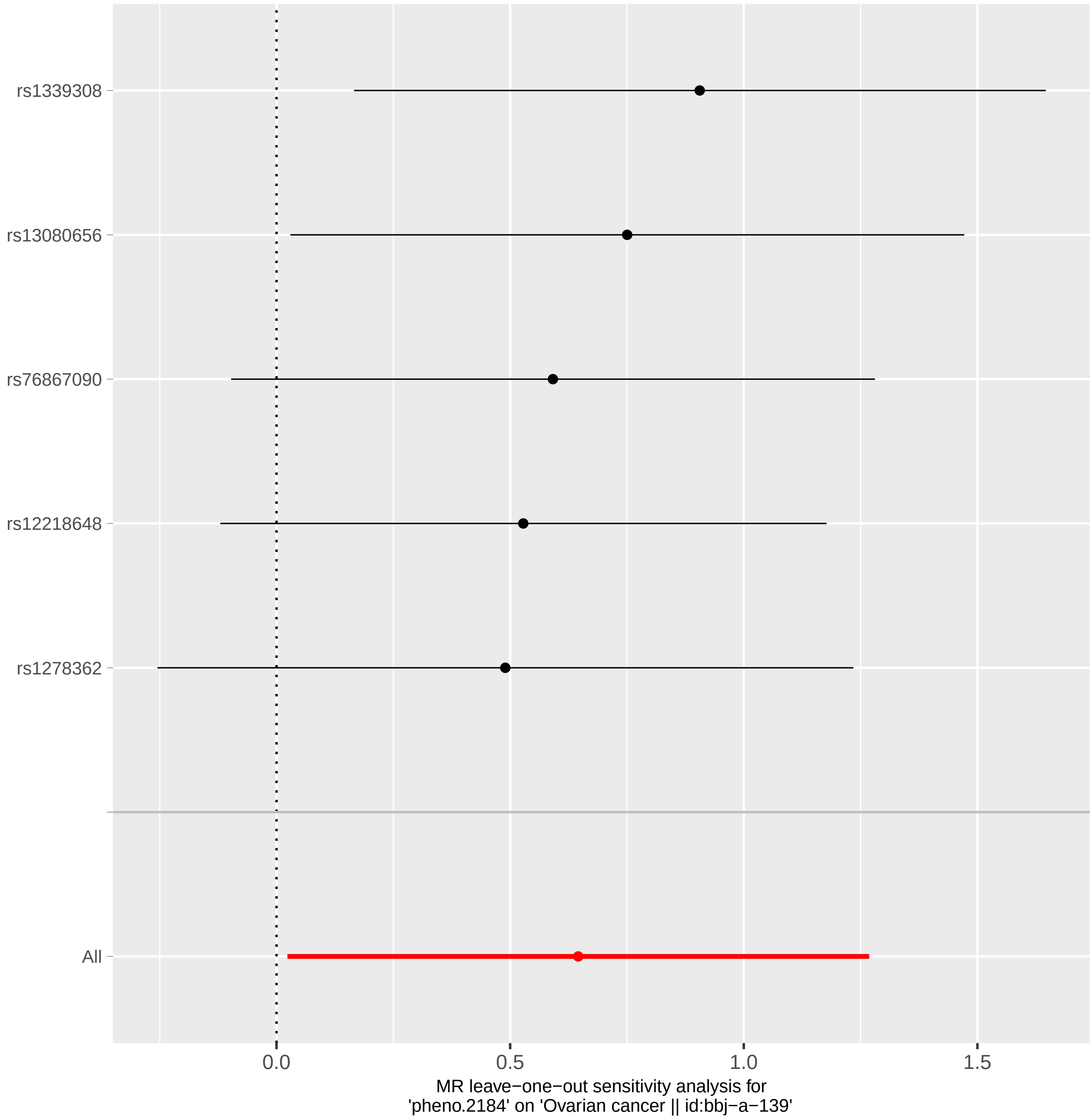
rs1339308
rs13080656
rs76867090
rs12218648
rs1278362
All
0.0
0.5
1.0
1.5
MR leave−one−out sensitivity analysis for
'pheno.2184' on 'Ovarian cancer || id:bbj−a−139'
